# Longitudinal Change of Psychological Distress among Healthcare Professionals with and without Psychological First Aid Training Experience during the COVID-19 Pandemic

**DOI:** 10.3390/ijerph182312474

**Published:** 2021-11-26

**Authors:** Hiroki Asaoka, Yuichi Koido, Yuzuru Kawashima, Miki Ikeda, Yuki Miyamoto, Daisuke Nishi

**Affiliations:** 1 Department of Psychiatric Nursing, Graduate School of Medicine, The University of Tokyo, 7-3-1 Hongo, Bunkyo-ku, Tokyo 1130033, Japan; s1010640@yahoo.co.jp (H.A.); yyuki@m.u-tokyo.ac.jp (Y.M.); 2 DMAT Secretariat, National Hospital Organization, 3256 Midoricho Tachikawa, Tokyo 1908579, Japan; koido@outlook.jp (Y.K.); yuzuru@dmat.jp (Y.K.); 3 College of Arts and Sciences, J. F. Oberlin University, 3758 Tokiwa-machi Machida-shi, Tokyo 1940294, Japan; mikicp@obirin.ac.jp; 4 Department of Mental Health, Graduate School of Medicine, The University of Tokyo, 7-3-1 Hongo, Bunkyo-ku, Tokyo 1130033, Japan; 5 Department of Public Mental Health Research, National Institute of Mental Health, National Center of Neurology and Psychiatry, 4-1-1 Ogawahigashicho, Kodaira, Tokyo 1878553, Japan

**Keywords:** psychological first aid, PFA, COVID-19, pandemic, healthcare professionals, psychological distress, depression

## Abstract

This study aimed to compare longitudinal change of the psychological distress of a group with psychological first aid (PFA) experience and a group without PFA experience among physicians and other healthcare professionals from before the novel coronavirus disease (COVID-19) pandemic to during the pandemic. The baseline survey was conducted in January 2020 (T1). The respondents in T1 were invited to participate in March (T2) and November 2020 (T3). Psychological distress was assessed by the Kessler 6 Scale. Participants were divided into two categories: a group with and a group without PFA experience. Participants were further divided between physicians and healthcare professionals other than physicians, because physicians are more likely to experience morally injurious events. A mixed-model repeated-measures ANOVA was conducted as an indicator of the group differences. In T1, 398 healthcare professionals participated. The longitudinal analysis of healthcare professionals other than physicians showed that psychological distress was significantly greater in the group without PFA experience than in the group with PFA experience (T1 vs. T3). This study showed psychological distress among healthcare professionals other than physicians was significantly greater in the group without PFA experience than in the group with PFA experience during the COVID-19 pandemic, but the results were not consistent among physicians.

## 1. Introduction

Since early 2020, the novel coronavirus disease (COVID-19) has spread throughout the world. Mental health problems have been reported among healthcare professionals responding to COVID-19 [1,2,3,4]. A systematic review reported an increased risk of mental health outcomes among healthcare professionals working in hospitals during a COVID-19 outbreak, with the prevalence of mental health problems being anxiety (23.2%), depression (22.8%), and insomnia (38.9%) [5]. Mental health problems have been reported to be associated with job performance, absenteeism, and turnover among healthcare professionals [6]. Prevention and countermeasures for mental health problems among healthcare professionals are important to maintain the healthcare system during COVID-19 outbreaks. 

A number of strategies have been recommended to support the mental health of healthcare professionals during COVID-19 outbreaks [7]. Some systematic reviews have been published on the effects of interventions, including psychological first aid (PFA) training for healthcare professionals to reduce adverse mental health sequelae during infectious disease outbreaks [8,9]. These studies showed PFA training may help improve not only the mental health of survivors, but also that of their supporters, such as healthcare professionals. PFA was originally developed to mitigate acute distress, and assess the need for continued mental healthcare through a compassionate and supportive presence for people in the immediate aftermath of a disaster or terrorism [10]. Several PFA frameworks and models, such as those from WHO and Johns Hopkins, are currently available for emergency management. For example, healthcare professionals in the UK can access a free PFA training course [11]. A previous randomized controlled trial reported on the mental health benefits of receiving PFA training for healthcare professionals who were frontline responders at various local and overseas disasters, and mass gathering events [12].

However, some systematic reviews concluded there was a lack of evidence that interventions, such as PFA, during or after disease epidemics and pandemics were beneficial to the resilience and mental health of healthcare professionals [13,14]. A previous study of a cluster-randomized trial investigated PFA training for burnout in healthcare professionals responding to the Ebola virus disease outbreak, and found no significant improvement in burnout in the intervention group [15]. Limitations of this previous study were that burnout was the only measured mental health outcome, and other mental health outcomes, such as psychological distress and depression, were not measured. In addition, a systematic review pointed that the effect of PFA training on burnout was uncertain because the certainty of the evidence of this previous study was very low [13]. Another previous study showed that an intervention combining PFA workshops and cognitive behavioral therapy significantly improved the stress, anxiety, depression, and anger domains among healthcare professionals in the Ebola treatment center following the 2014 Ebola virus disease outbreak in West Africa [16]. However, this study did not investigate the effect of PFA training alone on the mental health of healthcare professionals during the Ebola virus disease outbreak. Therefore, there are few studies that have investigated the effects of PFA training on the mental health of healthcare professionals during an emerging infectious disease pandemic.

In addition, previous studies have not revealed the target populations for which PFA training is effective [15,16], but it is important to identify such populations by characteristics such as differences in occupations among healthcare professionals. Mental health has been reported to vary by the occupation of healthcare professionals during the COVID-19 pandemic [17]. Due to challenging ethical scenarios and potential guilt generated by the inability to save patients’ lives, healthcare professionals are prone to be exposed to potentially morally injurious events and subsequent psychological distress during the COVID-19 pandemic. Some studies showed that physicians were more likely than other healthcare professionals to have poorer mental health during a severe acute respiratory syndrome outbreak and the COVID-19 pandemic [18,19]. Physicians are more likely than other healthcare professionals to experience morally injurious events because they must make life-and-death decisions about patients. A previous study, which explored the prevalence of potentially morally injurious events among physicians who worked in COVID-19 treatment medical units, showed that almost 50% of the participants experienced high levels of such exposure [20]. Therefore, it is important to examine the effects of PFA training on different occupations of healthcare professionals, especially comparing physicians with other healthcare professionals.

This study aimed to compare the longitudinal change in the psychological distress of a group with PFA training experience and a group without PFA training experience, comparing physicians with other healthcare professionals from before the pandemic (January 2020) to during the pandemic (November 2020) in Japan.

## 2. Materials and Methods

### 2.1. Participants

The Disaster Psychiatric Assistance Team (DPAT) and Disaster Medical Assistance Team (DMAT) are trained healthcare professionals who have the mobility to work in an acute phase of disasters. They are among the major disaster medical relief teams in Japan who respond at the onset of a disaster for a couple of days. DPAT and DMAT members (physicians, nurses, and other healthcare professionals, such as occupational therapists and pharmacists) usually work at their own hospital. The national or prefectural government requests a deployment to their hospitals at a time of need. The selected members provide rescue activities to the affected areas of the disaster or accident sites for several days, and return to their normal working hospital after the rescue activity. The recruited participants included DPAT and DMAT members who met the following inclusion criteria in this study: (1) native Japanese speaker or nonnative speaker with Japanese conversational abilities; (2) aged 18 years or older; (3) able to receive an e-mail of the written guide for this study from the DPAT office or the DMAT office; (4) physically and psychologically capable of understanding and providing consent for study participation.

### 2.2. Study Design

Healthcare professionals belonging to DPAT or DMAT were recruited for this internet-based cohort survey. The baseline survey of this study was conducted from 29 January to 21 February 2020 (T1). The respondents in T1 were invited to participate from 11 March to 2 April 2020 (T2) [1], and from 23 October to 20 November 2020 (T3). For DPAT members, a recruit mail for this study was posted to the mailing list by the DPAT office, and for DMAT members, by the DMAT office. The recruit mail contained a written explanation of this study and the URL of a web page containing a consent form for this study and a questionnaire. Participants accessed the URL, read an explanation of the study, and responded online to the consent form and the questionnaire. For reference, the number of confirmed cases of COVID-19 in Japan was 93 on 21 February (T1), 2381 on 2 April (T2), and 125,267 on 20 November 2020 (T3) [21]. 

We decided on the timing of conducting the surveys for the following reasons. We conducted a survey (T1) on the participants of our study before the outbreak of COVID-19 in Japan from January to February 2020. After the start of the survey (T1), the COVID-19 outbreak occurred, and the infection of COVID-19 spread in Japan. We conducted a survey (T2) on the same participants from March to April 2020 to investigate their mental health during the COVID-19 outbreak. Survey (T3) was conducted from October to November 2020 as a survey six months after the survey (T2).

This study was ethically approved by the research ethics committee of the National Hospital Organization Disaster Medical Center (No. 2019-19), and the research ethics committee of the Graduate School of Medicine and Faculty of Medicine at the University of Tokyo (No. 2019164NI-(1)(2)(3)). This study was also ethically approved to use the information of DPAT and DMAT by the Ministry of Health, Labor and Welfare of Japan. Informed consent was obtained by the participant reading an ethical document, and completing a consent form on the web page. This study was conducted in accordance with the Strengthening the Reporting of Observational Studies in Epidemiology statement [22].

### 2.3. Measurements

#### 2.3.1. Outcome: Psychological Distress

Psychological distress was assessed by the Kessler 6 Scale (K6). It is a self-report questionnaire designed to evaluate psychological distress, rating one’s condition for the prior month [23]. The K6 consists of six items and uses a five-point response format ranging from 0 (none of the time) to 4 (all of the time); the total score ranges from 0 to 24. Validity and reliability of the Japanese version have been confirmed [24]. We used K6 to measure psychological distress at T1, T2, and T3.

#### 2.3.2. Independent Variables

Items about PFA training experience were originally developed through discussion among healthcare professionals and researchers (HA, YKo, YKa, MI, YM, and DN) who were engaged in mental health among healthcare professionals or PFA in Japan. The question about the experience of taking PFA training was asked at T1: “Have you ever taken psychological first aid (PFA) training?”, and was answered by a binary (yes/no). 

Resilience was assessed by the Tachikawa Resilience Scale (TRS). TRS was developed on the basis of data obtained from unstructured interviews with Japanese motor vehicle accident survivors, and as a concise scale of resilience for Japanese populations [25]. TRS consists of 10 items and uses a 7-point response format ranging from 1 (strongly disagree) to 7 (strongly agree), with a total score ranging from 10 to 70. Higher scores reflect higher resilience. Validity and reliability of the Japanese version have been confirmed [25]. We used TRS to measure resilience at T1.

Demographic variables, such as sex, age, an affiliation of DMAT or DPAT, hospital affiliation, e-mail address, occupation, years of occupational experience, and years of DMAT or DPAT experience, were retrieved at T1 survey. Contact with a patient of COVID-19 at their own workplace was measured at T2 and T3. We did not survey about contact with a patient of COVID-19 at their own workplace at T1 because there were very few cases of COVID-19 in Japan at T1.

### 2.4. Statistical Analysis

We analyzed the dataset of participants who completed all questions of the self-report questionnaire. Participants were divided into two categories: a group with PFA training experience, and a group without PFA training experience. Participants were further divided into two groups according to their occupation: physicians, and healthcare professionals other than physicians. We used a chi-squared test to evaluate the difference in categorical variables in the two groups between physicians and healthcare professionals other than physicians, and used a Student’s *t*-test to evaluate the difference in continuous variables.

A mixed model repeated measures ANOVA with an unstructured covariance matrix was conducted using a group (group with PFA training experience vs. group without PFA training experience) × time (T1, T2, T3) interaction as an indicator of the group differences, adjusted for sex, years of DMAT or DPAT, and Tachikawa resilience scale (total score) [26,27,28]. We adjusted for resilience in the adjusted model because it has been reported as an associated factor of psychological distress in previous studies [27]. This model handled and imputed missing data with restricted maximum likelihood estimation, assuming missing at random. Therefore, we included all study participants who completed the baseline survey (N = 398) in our analyses by using the mixed model repeated measures ANOVA. Statistical significance was set as a two-sided *p* < 0.05. All analyses were conducted using SPSS version 26.0 J for Windows (SPSS, Tokyo, Japan). An adjustment for multiple comparisons was not made because all the statistical tests were conducted simultaneously in a regression model.

## 3. Results

At T1, of 9733 DMAT and DPAT members, 592 (response rate: 6.1%) agreed to participate in this study, and 476 (4.9%) completed all questions. After excluding 78 participants who were clerical workers and not healthcare professionals, the final sample comprised 398 (4.1%) healthcare professionals. Among the 398 participants in T1, 90 participants (follow up rate: 22.6%) answered at T2, and 80 participants (20.1%) answered at T3. The characteristics of participants are shown in Table 1. The mean age was 42.3 (SD = 8.2): 120 (30.2%) were physicians, and 278 (69.8%) were healthcare professionals other than physicians (mainly nurses). The number of participants with experience in PFA training was 88 (22.1%). The mean total score of TRS was 46.1 (SD = 8.7). Among the 90 participants at T2, 6 (6.7%) participants were contact with a patient of COVID-19 at their own workplace. Among the 80 participants at T3, 41 (51.2%) participants were contact with a patient of COVID-19 at their own workplace. In the results of the chi-squared test and the Student’s *t*-test between physicians and healthcare professionals other than physicians, there were significant differences in sex, age, years of occupational experience, and years of DMAT or DPAT experience. There were not significant differences in PFA training experience between the two groups.

The results of a mixed-model ANOVA are shown in Table 2. Among physicians, being in the group with PFA training experience was associated with a significant increase in psychological distress from T1 to T3 compared to being in the group without PFA training experience (crude estimates of fixed effect 4.02 [95% CI 0.44–7.60], *p* = 0.03). Among healthcare professionals other than physicians (mainly nurses), being in the group with PFA training experience was associated with a significant decrease in psychological distress from T1 to T3 compared to being in the group without PFA training experience (crude estimates of fixed effect −2.78 [95% CI −4.62–−0.95], *p* = 0.003).

The results of a mixed model ANOVA, adjusted for sex, years of DMAT or DPAT, and the TRS, are shown in Table 3. Among physicians, being in the group with PFA training experience was not associated with a significant increase in psychological distress from T1 to T2 and T3 compared to being in the group without PFA training experience. Among healthcare professionals other than physicians (mainly nurses), being in the group with PFA training experience was associated with a significant decrease in psychological distress from T1 to T3 compared to being in the group without PFA training experience (adjusted estimates of fixed effect −2.38 [95% CI −4.30–−0.46], *p* = 0.02). The estimated mean of psychological distress among physicians increased in both groups from T1 to T2, and increased in the group with PFA training experience and decreased in in the group without PFA training experience from T2 to T3. Among healthcare professionals other than physicians (mainly nurses), the estimated mean of psychological distress increased in both groups from T1 to T2, and decreased in the group with PFA training experience and increased in the group without PFA training experience from T2 to T3.

## 4. Discussion

This longitudinal study investigated the relationship between the experience of PFA training and psychological distress among healthcare professionals during the COVID-19 pandemic. The result of this longitudinal analysis among healthcare professionals other than physicians (mainly nurses) showed that psychological distress was significantly greater among those without PFA training experience than those with PFA training experience (T1 vs. T3). Among physicians, psychological distress was not significantly different between the group with PFA training experience and the group without PFA training experience in the adjusted model (T1 vs. T2, T3).

The result of this longitudinal analysis showed that psychological distress among healthcare professionals other than physicians (mainly nurses) increased from January (T1) to April (T2) 2020, but decreased in the group with PFA training experience and increased in the group without PFA training experience from April (T2) to November (T3), and psychological distress was significantly greater in the group without PFA training experience than in the group with PFA training experience (T1 vs. T3). In the period from January to April 2020, the first domestic case of COVID-19 was confirmed on 16 January; from there, the number of COVID-19 cases rapidly increased, and the first state of emergency was declared on 7 April in Japan [29]. The finding that psychological distress increased from January to April 2020 in both groups with and without PFA training experience was consistent with a previous study of healthcare professionals in Japan [30]. The psychological distress of the participants may have increased regardless of their previous PFA training experience due to their anxiety associated with the COVID-19 outbreak in Japan. In addition, the increased workload in the workplace associated with the COVID-19 outbreak may have also contributed to the increased psychological distress among the participants in this study [3]. From April to November 2020, the first emergency declaration was lifted on 25 May, and the number of COVID-19 cases subsequently fluctuated without a sharp increase until November 2020 [21]. Psychological distress decreased in the group with PFA training experience and increased in the group without PFA training experience from April to November, and there were different changes in psychological distress in the two groups. A previous randomized controlled trial reported on the mental health benefits of receiving PFA training on healthcare workers who are frontline responders for various local and overseas disasters and mass gathering events [12]. The study showed there was no change in general psychopathology in the intervention group, although controls showed increased general psychopathology over time. This result was consistent with the results of our study of the groups with and without PFA training experience from T2 to T3, although the participants’ responses differed between disasters and COVID-19. The reason for the difference between the group with PFA training and the group without PFA training might be that the training provides psychological benefits for the person delivering it, in addition to the recipients, as it teaches coping strategies, and facilitates connection and linkage to support systems and services that can be used to protect the self, as well as to support others. In addition, PFA is a generic disaster relief program that can be implemented either during or immediately after a disaster, and can apply to anyone impacted by the event [31]. Participants who experienced PFA training may have been able to provide better treatment and care to COVID-19 patients during the COVID-19 pandemic, thus preventing their own mental health from deteriorating. A possible explanation for the different changes in psychological distress between January (T1) and April (T2) 2020, and between April (T2) and November (T3) 2020 is that the participants might not know how to deal with COVID-19 patients in the early stages of the COVID-19 pandemic, but they might gradually become able to apply PFA training to the treatment and care of COVID-19 patients. This study showed that psychological distress was significantly greater in the group without PFA training experience than in the group with PFA training experience (T1 vs. T3). The results suggest that among healthcare professionals other than physicians (mainly nurses), PFA training experience may be an effective approach to prevent mental health problems for those responding to COVID-19 patients during the COVID-19 pandemic.

Among physicians, psychological distress increased in both groups from January (T1) to April (T2) 2020, and increased in the group with PFA training experience and decreased in the group without PFA training experience from April (T2) to November (T3). Psychological distress was not significantly different between the group with PFA training experience and the group without PFA training experience in the adjusted model (T1 vs. T2, T3). The psychological distress among physicians in both groups may have increased due to their anxiety associated with the COVID-19 outbreak and the increased workload in the workplace in the wake of the COVID-19 pandemic from January to April 2020, as well as in healthcare professionals other than physicians (mainly nurses). From April to November, psychological distress was not significantly changed among physicians in the group with PFA training experience, but decreased in the group without PFA training experience in the adjusted model, which was different from the results of healthcare professionals other than physicians (mainly nurses). Potential risks from delivering PFA training were reported, such as confusion about role boundaries, inadequate skills development, and inconsistent use of PFA guidance, which could potentially harm both a person in distress and PFA providers [32,33]. PFA providers helping hurricane survivors reported that they often hesitated or over-estimated their roles in responding to survivors’ needs [32]. Findings from a PFA training tailored to dealing with an outbreak of Ebola virus described potential risks, for example, giving false reassurance to calm a distressed person by promising that everything would be fine, which undermined their credibility and was counterproductive [33]. In addition, they also found that trainees attempted to solve the problem for a distressed person rather than promoting the person’s own coping abilities, which could decrease self-efficacy and, ultimately, hinder recovery. As in the results of these other research studies, the experience of PFA training among physicians may have been a risk factor for psychological distress during the COVID-19 pandemic in this study. These results suggest that additional psychosocial interventions and mental health care may be important for physicians during the COVID-19 pandemic, in addition to PFA training. A previous study reported almost 50% of physicians who worked in COVID-19 treatment medical units experienced potentially morally injurious events [20], thus, it may be important to provide training on moral injury, and to discuss it with colleagues at the workplace.

In addition, psychological distress among physicians was not significantly different between the group with PFA training experience and the group without PFA training experience in the adjusted model, adjusted for sex, year of DMAT or DPAT, and Tachikawa resilience scale (T1 vs. T2, T3). The results suggest that gender, years of occupational experience, and resilience may have been associated with psychological distress among participants of this study, as shown in previous studies [26,27,28]. Among those variables, in a previous study, resilience was reported to improve after taking PFA training [34]. In the future, it may be important to investigate the relationship between the PFA training experience and resilience, and its relationship to mental health among healthcare professionals during the COVID-19 pandemic.

The result of this longitudinal analysis for healthcare professionals other than physicians (mainly nurses) showed that psychological distress was significantly greater among those without PFA training experience than in the group with PFA training experience (T1 vs. T3). However, psychological distress among physicians was not significantly different between the group with PFA training experience and the group without PFA training experience in the adjusted model (T1 vs. T2, T3). The reason why the results differed by occupation may be related to the nature of the work. Since healthcare professionals other than physicians were mainly responsible for non-critical decision-making tasks, such as patient care during the COVID-19 pandemic, they were able to apply the content of the PFA training to their daily work. On the other hand, physicians have to make important decisions about patients and be extra vigilant when examining and reviewing patients during the COVID-19 pandemic. Previous studies showed that the nature of their work worsened the mental health of physicians more than it did for other healthcare professionals during a severe acute respiratory syndrome outbreak and the COVID-19 pandemic [18,19]. Since physicians were mainly responsible for important decision-making, such as deciding on treatment plans for patients, and examining patients during the COVID-19 pandemic, the content of the PFA training may not have been applicable to their daily work. In addition, because physicians had improved skills and abilities to understand patients’ feelings by taking PFA training, they had more opportunities to be morally injured by empathizing with patients’ feelings during the COVID-19 pandemic, which may have worsened the psychological distress of participants with PFA training experience. This study demonstrated the effectiveness of PFA training on psychological distress for healthcare professionals other than physicians during the COVID-19 pandemic, but not for physicians, suggesting that caution may be needed in providing PFA training to physicians. Further research is needed to understand the effectiveness of PFA training on mental health for different occupations of healthcare professionals.

## 5. Limitations of the Study

This study has several limitations. Firstly, the participants were not asked when they took the PFA training, so we do not know how long before January 2020 they might have taken it. Participants with PFA training experience gained different knowledge and skills depending on when they attended PFA training, which may have different effects on participants’ psychological distress during the COVID-19 pandemic. Second, the response rate was low, which may limit the external validity of this study. Non-responders could have been too stressed to respond or not at all stressed, and, therefore, not interested in this survey. Additionally, the low response and follow up rates may have caused biases in the analysis. Due to this limitation of this study, in order to show the association between experience of PFA training and psychological distress during the COVID-19 pandemic, it is necessary to investigate in the study with higher response and follow up rates. Third, motivated healthcare professionals tend to register as DPAT and DMAT members in general; thus, DPAT and DMAT members are not representative of healthcare professionals in Japan. Finally, this study was an observational study; in the future, interventional studies, such as randomized controlled trials, would be necessary to show reliable results.

## 6. Conclusions

This longitudinal study investigated the relationship between the experience of PFA training and psychological distress among healthcare professionals during the COVID-19 pandemic. The result of this longitudinal analysis showed that psychological distress among healthcare professionals other than physicians (mainly nurses) increased from January (T1) to April (T2) 2020, and decreased in the group with PFA training experience and increased in the group without PFA training experience from April (T2) to November (T3), and psychological distress was significantly greater among those without PFA training experience than in the group with PFA training experience (T1 vs. T3). Among physicians, psychological distress increased in both groups from T1 to T2, and increased in the group with PFA training experience and decreased in the group without PFA training experience from T2 toT3, and psychological distress was not significantly different between the group with PFA training experience and the group without PFA training experience in the adjusted model (T1 vs. T2, T3). In the future, further research, especially an interventional study, is needed to show reliable results of the effectiveness of PFA training on mental health for different occupations of healthcare professionals.

## Figures and Tables

**Table 1:** Demographic characteristics of participants (*n* = 398).

	All Participants (*n* = 398)				Physicians (*n* = 120)				Health Care Professionals Other than Physicians (*n* = 278)				*p* ^a^
	*n*	%	Mean	SD	*n*	%	Mean	SD	*n*	%	Mean	SD	
Sex													<0.01
Men	287	72.1			108	90.0			179	64.4			
Women	111	27.9			12	10.0			99	35.6			
DMAT or DPAT													0.37
DMAT	315	79.1			100	83.3			215	77.3			
DPAT	77	19.3			19	15.9			58	20.9			
DMAT & DPAT	6	1.6			1	0.8			5	1.8			
Age (years)			42.3	8.2			46.4	7.6			40.5	7.8	<0.01
Occupational experience (years)			18.0	7.9			20.2	7.6			17.0	7.9	<0.01
DMAT or DPAT experience (years)			4.9	4.1			6.4	4.3			4.3	3.8	<0.01
PFA training experience (yes)	88	22.1			21	17.5			67	24.1			0.15
K6: T1 (range: 0–24) ^b^			3.0	3.6			3.0	3.5			3.0	3.6	0.91
K6: T2 (range: 0–24) ^c^			3.7	4.3			4.3	4.9			3.1	3.9	0.19
K6: T3 (range: 0–24) ^d^			3.4	4.2			3.1	3.7			3.5	4.4	0.69
TRS (range: 10–70) ^e^			46.1	8.7			46.4	9.7			46.0	8.2	0.67

SD, standard deviation. PFA, psychological first aid. K6, the Kessler 6 Scale. K6 is a self-report questionnaire designed to evaluate psychological distress. TRS, Tachikawa resilience scale. TRS was developed as a concise scale of resilience for Japanese populations. Sex, DMAT or DPAT, age, occupation, occupational experience, DMAT or DPAT experience, PFA training experience, and TRS were measured at T1. ^a^ We used a chi-squared test to evaluate the difference in categorical variables in the two groups, and used a Student’s *t*-test to evaluate the difference in continuous variables. ^b^ Mean of K6 among 398 participants on T1. ^c^ Mean of K6 among 90 participants on T2 out of 398 respondents who had participated in T1. ^d^ Mean of K6 among 80 participants on T3 out of 398 respondents who had participated in T1. ^e^ Mean of TRS among 343 participants on T1, excluding 55 participants who did not respond to the TRS.

**Table 2:** The crude estimated mean of psychological distress ^a^ at baseline (T1), T2, and T3 during the COVID-19 pandemic among a cohort of Japanese healthcare professionals: the mixed model with repeated measures (N = 398).

	Physicians (*n* = 120)			Healthcare Professionals Other than Physicians (Mainly Nurses, *n* = 278)		
Survey (Time of Survey)	Group without PFA Training Experience ^b^ (*n* = 99)	Group with PFA Training Experience ^b^ (*n* = 21)	Survey x Group Interaction	Group without PFA Training Experience ^b^ (*n* = 211)	Group with PFA Training Experience ^b^ (*n* = 67)	Survey x Group Interaction
	Estimated Mean (SE)	Estimated Mean (SE)	*p* Value	Estimated Mean (SE)	Estimated Mean (SE)	*p* Value
T1 (February 2020)	3.0 (0.4)	3.0 (0.8)	ref	3.0 (0.3)	2.8 (0.5)	ref
T2 (March 2020)	4.0 (0.7)	5.9 (1.5)	0.26 (T1 vs. T2)	3.5 (0.6)	3.0 (0.8)	0.76 (T1 vs. T2)
T3 (October 2020)	2.5 (0.7)	6.6 (1.6)	0.03 * (T1 vs. T3)	4.1 (0.5)	1.1 (0.8)	0.003 ** (T1 vs. T3)

COVID-19: Coronavirus disease 2019. SE: standard error. * *p* < 0.05, ** *p* < 0.001. ^a^ Psychological distress was measured by K6. The scores ranged from 0 to 24, with the higher scores being indicative of higher distress. ^b^ The information about PFA training experience was measured at T1.

**Table 3:** The adjusted estimated mean of psychological distress ^a^ at baseline (T1), T2, and T3 during the COVID-19 pandemic among a cohort of Japanese healthcare professionals: the mixed model with repeated measures (N = 343 ^b^).

	Physicians (*n* = 104)			Healthcare Professionals Other than Physicians (Mainly Nurses, *n* = 239)		
Survey (Time of Survey)	Group without PFA Training Experience ^c^ (*n* = 85)	Group with PFA Training Experience ^c^ (*n* = 19)	Survey x Group Interaction	Group without PFA Training Experience ^c^ (*n* = 178)	Group with PFA Training Experience ^c^ (*n* = 61)	Survey x Group Interaction
	Estimated Mean (SE)	Estimated Mean (SE)	*p* Value	Estimated Mean (SE)	Estimated Mean (SE)	*p* Value
T1 (February 2020)	4.1 (0.9)	3.4 (1.4)	ref	2.9 (0.5)	2.9 (0.7)	ref
T2 (March 2020)	5.1 (1.1)	5.8 (1.9)	0.45 (T1 vs. T2)	3.5 (0.7)	3.3 (0.9)	0.89 (T1 vs. T2)
T3 (October 2020)	3.8 (1.1)	6.3 (2.0)	0.11 (T1 vs. T3)	3.8 (0.7)	1.4 (0.9)	0.02 * (T1 vs. T3)

COVID-19: coronavirus disease 2019. SE: standard error. * *p* < 0.05. Adjusted for sex, year of DMAT or DPAT, Tachikawa resilience scale (total score). The Tachikawa resilience scale was developed as a concise scale of resilience for Japanese populations. ^a^ Psychological distress was measured by K6. The scores ranged from 0 to 24, with the higher scores being indicative of higher distress. ^b^ Among the 398 participants, 55 participants who did not respond to the TRS on T1 were excluded. ^c^ The information about PFA training experience was measured at T1.

## Data Availability

The data that support the findings of this study are available from the corresponding author, D.N., upon reasonable request. The data are not publicly available due to privacy reasons.

## References

[1] Asaoka H., Koido Y., Kawashima Y., Ikeda M., Miyamoto Y., Nishi D. (2020). Post-traumatic stress symptoms among medical rescue workers exposed to COVID-19 in Japan. Psychiatry Clin. Neurosci..

[2] Jerg-Bretzke L., Kempf M., Jarczok M.N., Weimer K., Hirning C., Gündel H., Erim Y., Morawa E., Geiser F., Hiebel N. (2021). Psychosocial Impact of the COVID-19 Pandemic on Healthcare Workers and Initial Areas of Action for Intervention and Prevention—The egePan/VOICE Study. Int. J. Environ. Res. Public Health.

[3] Kisely S., Warren N., McMahon L., Dalais C., Henry I., Siskind D. (2020). Occurrence, prevention, and management of the psychological effects of emerging virus outbreaks on healthcare workers: Rapid review and meta-analysis. BMJ.

[4] Marvaldi M., Mallet J., Dubertret C., Moro M.R., Guessoum S.B. (2021). Anxiety, depression, trauma-related, and sleep disorders among healthcare workers during the COVID-19 pandemic: A systematic review and meta-analysis. Neurosci. Biobehav. Rev..

[5] Pappa S., Ntella V., Giannakas T., Giannakoulis V.G., Papoutsi E., Katsaounou P. (2020). Prevalence of depression, anxiety, and insomnia among healthcare workers during the COVID-19 pandemic: A systematic review and meta-analysis. Brain Behav. Immun..

[6] Johnston D., Harvey S., Glozier N., Calvo R., Christensen H., Deady M. (2019). The relationship between depression symptoms, absenteeism and presenteeism. J. Affect. Disord..

[7] World Health Organization (2020). Mental Health and Psychosocial Considerations during the COVID-19 Outbreak. https://www.who.int/publications/i/item/WHO-2019-nCoV-MentalHealth-2020.1.

[8] Gross J.V., Mohren J., Erren T.C. (2021). COVID-19 and healthcare workers: A rapid systematic review into risks and preventive measures. BMJ Open.

[9] Muller A.E., Hafstad E.V., Himmels J.P.W., Smedslund G., Flottorp S., Stensland S.Ø., Stroobants S., Van De Velde S., Vist G.E. (2020). The mental health impact of the covid-19 pandemic on healthcare workers, and interventions to help them: A rapid systematic review. Psychiatry Res..

[10] Everly G.S., Flynn B.W. (2006). Principles and practical procedures for acute psychological first aid training for personnel without mental health experience. Int. J. Emerg. Ment. Health Hum. Resil..

[11] Gov. UK (2020). Psychological First Aid in Emergencies Training for Frontline Staff and Volunteers. https://www.gov.uk/government/news/psychological-first-aid-in-emergencies-training-for-frontline-staff-and-volunteers.

[12] Cheung Y.L. (2014). Psychological First Aid as a Public Health Disaster Response Preparedness Strategy for Responders in Critical Incidents and Disasters [Dissertation on the Internet].

[13] Pollock A., Campbell P., Cheyne J., Cowie J., Davis B., McCallum J., McGill K., Elders A., Hagen S., McClurg D. (2020). Interventions to support the resilience and mental health of frontline health and social care professionals during and after a disease outbreak, epidemic or pandemic: A mixed methods systematic review. Cochrane Database Syst. Rev..

[14] Yue J.-L., Yan W., Sun Y.-K., Yuan K., Su S.-Z., Han Y., Ravindran A.V., Kosten T., Everall I., Davey C.G. (2020). Mental health services for infectious disease outbreaks including COVID-19: A rapid systematic review. Psychol. Med..

[15] Sijbrandij M., Horn R., Esliker R., O’May F., Reiffers R., Ruttenberg L., Stam K., De Jong J., Ager A. (2020). The Effect of Psychological First Aid Training on Knowledge and Understanding about Psychosocial Support Principles: A Cluster-Randomized Controlled Trial. Int. J. Environ. Res. Public Health.

[16] Waterman S., Hunter E.C.M., Cole C.L., Evans L.J., Greenberg N., Rubin G.J., Beck A. (2018). Training peers to treat Ebola centre workers with anxiety and depression in Sierra Leone. Int. J. Soc. Psychiatry.

[17] Shaukat N., Ali D.M., Razzak J. (2020). Physical and mental health impacts of COVID-19 on healthcare workers: A scoping review. Int. J. Emerg. Med..

[18] Chan A.O.M. (2004). Psychological impact of the 2003 severe acute respiratory syndrome outbreak on health care workers in a medium size regional general hospital in Singapore. Occup. Med..

[19] Rossi R., Socci V., Pacitti F., Di Lorenzo G., Di Marco A., Siracusano A., Rossi A. (2020). Mental Health Outcomes Among Frontline and Second-Line Health Care Workers During the Coronavirus Disease 2019 (COVID-19) Pandemic in Italy. JAMA Netw. Open.

[20] Maftei A., Holman A.-C. (2021). The prevalence of exposure to potentially morally injurious events among physicians during the COVID-19 pandemic. Eur. J. Psychotraumatol..

[21] Ministry of Health, Labor and Welfare in Japan (2021). Data on New Coronavirus Infection in Japan. https://www.mhlw.go.jp/stf/seisakunitsuite/bunya/0000121431_00086.html.

[22] Von Elm E., Altman D.G., Egger M., Pocock S.J., Gotzsche P.C., Vandenbroucke J.P., STROBE Initiative (2014). The Strengthening the Reporting of Observational Studies in Epidemiology (STROBE) Statement: Guidelines for reporting observational studies. Int. J. Surg..

[23] Kessler R.C., Andrews G., Colpe L.J., Hiripi E., Mroczek D.K., Normand S.-L., Walters E.E., Zaslavsky A.M. (2002). Short screening scales to monitor population prevalences and trends in non-specific psychological distress. Psychol. Med..

[24] Furukawa T., Kessler R.C., Slade T., Andrews G. (2003). The performance of the K6 and K10 screening scales for psychological distress in the Australian National Survey of Mental Health and Well-Being. Psychol. Med..

[25] Nishi D., Uehara R., Yoshikawa E., Sato G., Ito M., Matsuoka Y. (2013). Culturally sensitive and universal measure of resilience for Japanese populations: Tachikawa Resilience Scale in comparison with Resilience Scale 14-item version. Psychiatry Clin. Neurosci..

[26] Jorm A.F., Windsor T.D., Dear K.B.G., Anstey K.J., Christensen H., Rodgers B. (2005). Age group differences in psychological distress: The role of psychosocial risk factors that vary with age. Psychol. Med..

[27] Preti E., Di Mattei V., Perego G., Ferrari F., Mazzetti M., Taranto P., Di Pierro R., Madeddu F., Calati R. (2020). The Psychological Impact of Epidemic and Pandemic Outbreaks on Healthcare Workers: Rapid Review of the Evidence. Curr. Psychiatry Rep..

[28] Song X., Fu W., Liu X., Luo Z., Wang R., Zhou N., Yan S., Lv C. (2020). Mental health status of medical staff in emergency departments during the Coronavirus disease 2019 epidemic in China. Brain Behav. Immun..

[29] Looi M.-K. (2020). Covid-19: Japan ends state of emergency but warns of “new normal”. BMJ.

[30] Sasaki N., Asaoka H., Kuroda R., Tsuno K., Imamura K., Kawakami N. (2021). Sustained poor mental health among healthcare workers in COVID-19 pandemic: A longitudinal analysis of the four-wave panel survey over 8 months in Japan. J. Occup. Health.

[31] Hooper J.J., Saulsman L., Hall T., Waters F. (2021). Addressing the psychological impact of COVID-19 on healthcare workers: Learning from a systematic review of early interventions for frontline responders. BMJ Open.

[32] Chandra A., Kim J., Pieters H.C., Tang J., McCreary M., Schreiber M., Wells K. (2014). Implementing Psychological First-Aid Training for Medical Reserve Corps Volunteers. Disaster Med. Public Health Prep..

[33] Horn R., O’May F., Esliker R., Gwaikolo W., Woensdregt L., Ruttenberg L., Ager A. (2019). The myth of the 1-day training: The effectiveness of psychosocial support capacity-building during the Ebola outbreak in West Africa. Glob. Ment. Health.

[34] Wang L., Norman I., Xiao T., Li Y., Leamy M. (2021). Psychological First Aid Training: A Scoping Review of Its Application, Outcomes and Implementation. Int. J. Environ. Res. Public Health.

